# Smoking cessation and influenza vaccination can reduce the healthcare burden of COPD

**DOI:** 10.18332/tid/167962

**Published:** 2023-08-26

**Authors:** Hui-Chuan Chang, Shih-Feng Liu, Ho-Chang Kuo, Kuang-Den Chen, Jui-Fang Liu, Ching-Wan Tseng, Ching-Mei Weng, Teng-Ching Chou

**Affiliations:** 1 Department of Respiratory Therapy, Kaohsiung Chang Gung Memorial Hospital, Kaohsiung, Taiwan; 2 Division of Pulmonary and Critical Care Medicine, Department of Internal Medicine, Kaohsiung Chang Gung Memorial Hospital, Kaohsiung, Taiwan; 3 College of Medicine, Chang Gung University, Taoyuan, Taiwan; 4 Department of Pediatrics, Kaohsiung Chang Gung Memorial Hospital, Kaohsiung, Taiwan; 5 Institute of Translation Research in Biomedicine, Liver Transplantation Center and Department of Surgery, Kaohsiung Chang Gung Memorial Hospital, Kaohsiung, Taiwan; 6 College of Medicine, Chang Gung University, Kaohsiung, Taiwan; 7 Department of Respiratory Care, Chang Gung University of Science and Technology, Chiayi, Taiwan; 8 Chronic Diseases and Health Promotion Research Center, Chang Gung University of Science and Technology, Chiayi, Taiwan

**Keywords:** smoking cessation, influenza vaccination, COPD, hospital utilization, emergency utilization

## Abstract

**INTRODUCTION:**

Influenza vaccination (INV) and smoking cessation (SC) have individual positive effects on COPD, but their synergistic impact has yet to be extensively studied. This retrospective study aimed to assess the combined effect of SC and IV on the medical burden of COPD, including medical visits, hospitalization, medical expenses, and the occurrence of respiratory failure.

**METHODS:**

Patients with COPD who visited our medical center between January and October 2018 were included in the study. The patients were categorized into four groups: Group I (no SC or INV), Group II (INV only), Group III (SC only), and Group IV (both SC and INV). The outcomes analyzed were emergency utilization, hospital utilization, and occurrence of respiratory failure. Airflow limitation was stratified according to GOLD guidelines, and successful smoking cessation was defined as not smoking for at least one year.

**RESULTS:**

A total of 357 patients were included in the study. Group I (119 patients) neither smoking cessation nor influenza vaccination; Group II (66 patients) had only influenza vaccination; Group III (94 patients), had only smoking cessation, Group IV (78 patients), with both smoking cessation and influenza vaccination. Group IV had lower odds of emergency utilization (OR=0.13; 95% CI: 0.07–0.25), hospital utilization (OR=0.13; 95% CI: 0.05–0.30, p<0.001), and occurrence of respiratory failure (OR=0.13; 95% CI: 0.04–0.40, p<0.001).

**CONCLUSIONS:**

Combined smoking cessation and influenza vaccination are more effective in reducing the medical burden of COPD compared to either intervention alone or neither. These findings highlight the importance of promoting both smoking cessation and influenza vaccination in the management of COPD.

## INTRODUCTION

The prevalence of chronic obstructive pulmonary disease (COPD) is increasing worldwide, leading to higher mortality rates. Additionally, the burden of chronic conditions associated with ageing and smoking is expected to further rise^1^. In Taiwan, respiratory diseases accounted for approximately 9.75% of national health insurance costs in 2020, with medication expenses constituting the largest portion of the medical budget.

Cigarette smoking profoundly impacts chronic inflammation in COPD, exacerbating inflammation, promoting infections, causing tissue damage, and potentially contributing to allergy development^2^. Smoking is responsible for 85–90% of COPD cases in developed economies and increases the risk of COPD-related death by 13 times^3,4^. Thus, smoking is recognized as one of the most crucial factors influencing prognosis, symptom severity, and disease progression^5^.

Influenza is a global disease that annually causes significant morbidity and mortality. The occurrence of influenza infection in COPD patients is much higher compared to non-COPD individuals^6^. Influenza vaccination has demonstrated its ability to reduce outpatient visits, influenza-related hospitalizations, mortality rates, and complications associated with influenza^7,8^. Furthermore, influenza vaccination has shown greater effectiveness in reducing hospital utilization, emergency room visits, and the incidence of respiratory failure in COPD patients with moderate, severe, and very severe airflow obstruction compared to those with mild obstruction^9^. Consequently, influenza vaccination is routinely recommended for COPD patients in many countries, including Taiwan.

It is well established that smoking is associated with an increased prevalence and severity of respiratory infections, including a higher susceptibility to pandemic influenza^10-12^. Smoking is also consistently linked to a greater risk of hospitalization due to influenza infection^13^. However, previous studies have observed that smoking cessation has immediate effects in reducing costs and healthcare utilization in COPD patients, regardless of age, duration of COPD, or the presence of comorbidities^14^. Similar to influenza vaccination, smoking cessation has a positive impact on COPD by decreasing hospitalizations and emergency department utilization^15,16^.

This study aims to investigate whether the combination of smoking cessation and influenza vaccination can result in lower medical costs associated with COPD compared to either intervention alone or no intervention.

## METHODS

### Study design

**Figure 1:**
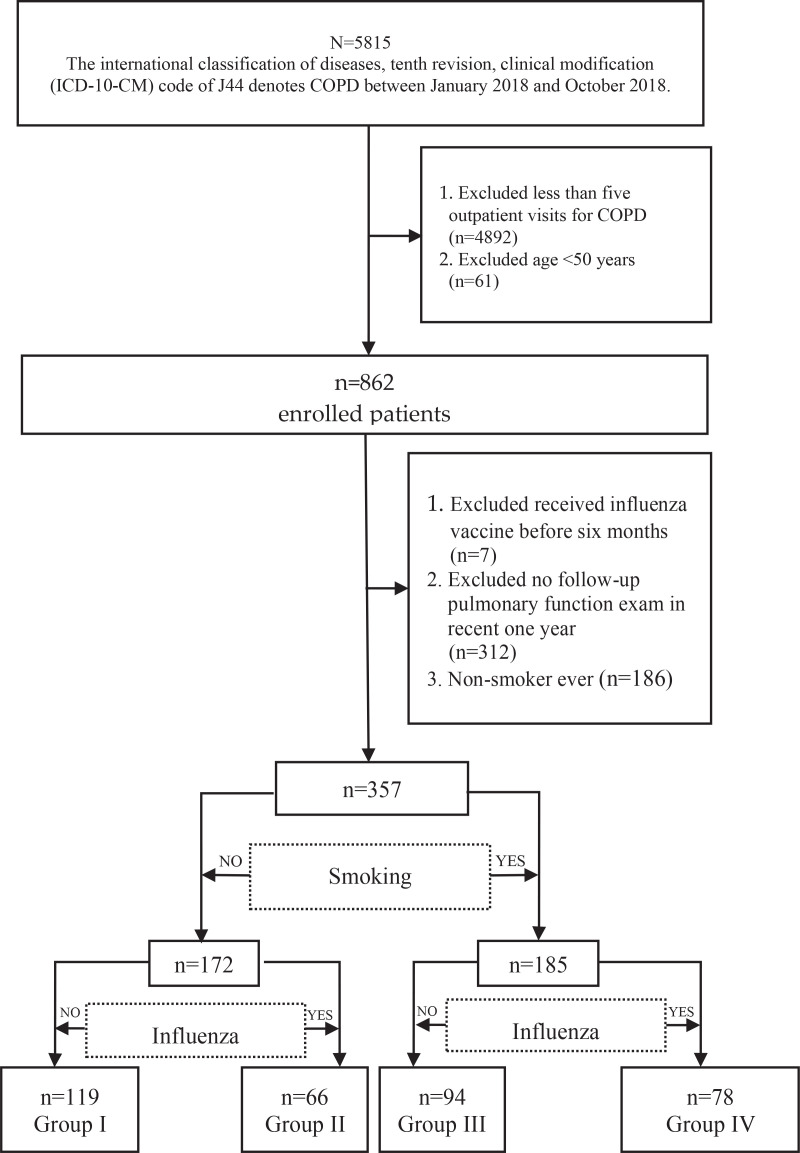
Flowchart of the study design and patient selection from Chang Gung Memorial Medical Center database. Group I (no smoking cessation or influenza vaccination), Group II (influenza vaccination only), Group III (smoking cessation only), and Group IV (both smoking cessation and influenza vaccination)

This retrospective study analyzed patients diagnosed with COPD between January and October 2018 using the Kaohsiung Chang Gung Memorial Medical Center database. Participant data were tracked until 30 September 2019, to explore the impact of influenza vaccination and smoking cessation on healthcare resource utilization, including emergency department visits, hospitalization, and associated costs. (Figure 1).

### Study participants

The data included primary diagnosis codes from the International Classification of Diseases, Tenth Revision Clinical Modification (ICD-10) J44, obtained from the Kaohsiung Chang Gung Memorial Medical Center database. A total of 5818 patients were initially enrolled, with those having fewer than five outpatient visits within one year excluded based on COPD Surveillance data, which showed an average of 4.9 outpatient visits annually over a six-year period^17^. This ensured that enrolled patients were regularly followed up. Additionally, patients aged <50 years were excluded, and to minimize the interaction effects of previous influenza vaccinations, those who received influenza vaccinations within the past year and those who did not undergo pulmonary function examination in the last year, were excluded. In this study, we defined individuals who successfully ceased smoking for at least one year as ex-smokers, and those without successful smoking cessation as current smokers, as confirmed by chart records. Patients with no history of smoking were excluded. Finally, a total of 357 patients were included in this study and were divided into four groups: Group I consisted of individuals who neither underwent smoking cessation nor received influenza vaccination; Group II included current smokers who received an influenza vaccination; Group III comprised ex-smokers who did not receive an influenza vaccination; and Group IV consisted of ex-smokers who received an influenza vaccination.

### Clinical variables

Demographic data were collected following influenza vaccination over a one-year period. The recorded variables included sex, age, smoking cessation status for at least one year, influenza vaccination status, results of pulmonary function tests, utilization of emergency department visits and hospitalizations, length of hospital stay, analysis of medical expenditure, and occurrence of respiratory failure.

### Statistical analysis

Continuous variables are presented as mean ± standard deviation, while categorical variables are expressed as absolute values and percentages. Statistical significance was set at p<0.05. Chi-squared tests were used for categorical variables, and Student’s t-test was used for continuous variables. The categorical variables (Groups I–IV) were based on whether participants received influenza vaccination and successful smoking cessation. For the analysis of emergency visits, hospital admissions, length of hospital stay, and hospitalization costs, medians and interquartile ranges (25th percentile, p25, and 75th percentile, p75) were calculated, Kruskal-Wallis test and multivariate logistic regression analysis were performed to assess the association of each categorical variable with emergency department utilization, hospital utilization, and the occurrence of respiratory failure. Data were analyzed using STATA version 12 (College Station, TX, USA). A two-sided p<0.05 was considered statistically significant.

## RESULTS

### Demographic characteristics of the study population

Table 1 presents the demographic characteristics of the study population. Out of the 357 enrolled patients, 13.73% were aged 50–59 years, 30.53% were aged 60–69 years, 35.85% were aged 70–79 years, and 19.89% were aged ≥80 years. Among the participants, 92.44% were males. Groups I, II, III, and IV comprised 119, 66, 94, and 78 patients, respectively. There was a higher prevalence of patients in the 60–69 years and 70–79 years age cohorts (p=0.253), and males had a higher prevalence than females in each group (p=0.126). There were no significant differences in FVC (% of predicted value), FEV1/FVC (%), and FEV1 (% of predicted value) between the groups.

**Table 1:** Baseline characteristics of the study population among groups. Group I (no smoking cessation or influenza vaccination), Groups II (influenza vaccination only), Group III (smoking cessation only) and Group IV (both smoking cessation and influenza vaccination)

*Variables*	*Total (N=357) n (%)*	*Group I (N=119) n (%)*	*Group II (N=66) n (%)*	*Group III (N=94) n (%)*	*Group IV (N=78) n (%)*	*p*
**Age** (years)						0.253
50–59	49 (13.73)	12 (10)	7 (10.61)	14 (14.89)	16 (20.51)	
60–69	109 (30.53)	40 (33.61)	14 (21.21)	37 (39.36)	18 (23.08)	
70–79	128 (35.85)	43 (36.13)	28 (42.42)	29 (30.85)	28 (35.90)	
≥80	71 (19.89)	24 (20.17)	17 (25.76)	14 (14.89)	16 (20.51)	
**Sex**						0.126
Female	27 (7.56)	14 (11.76)	5 (7.58)	3 (3.19)	5 (6.41)	
Male	330 (92.44)	105 (88.24)	61 (92.42)	91 (96.81)	73 (95.59)	
**Smoking cessation**						
Success	185 (51.82)			√	√	
Failure	172 (48.18)	√	√			
**Influenza vaccination**						
Received	213 (59.66)		√		√	
Not received	144 (40.34)	√		√		
**Pulmonary function test**, mean (SD)						
FVC (% of predicted value)	75.69	71.98 (31.82)	79.25 (23.79)	81.05 (18.40)	73.76 (20.79)	0.170
FEV1/FVC (%)	54.95	55.06 (14.67)	55.00 (9.99)	55.39 (15.10)	54.44 (10.36)	0.328
FEV1 (% of predicted value)	54.49	53.63 (30.98)	55.11 (13.79)	56.98 (19.52)	53.19 (18.60)	0.260

### Comparison of medical utilization among Groups I–IV

Table 2 presents the medical utilization comparison among Groups I–IV. Compared to Group I, Groups II, III, and IV had significantly fewer emergency visits (p<0.001), hospital admissions (p<0.001), length of hospital stay (p<0.001), and hospitalization costs (p<0.001). When comparing Group IV to Group II, there were no significant differences in emergency visits (p=0.767), hospital admissions (p=0.478), length of hospital stay (p=0.159), and hospitalization costs (p=0.365). When comparing Group IV to Group III, there were no significant differences in emergency visits (p=0.115). Still, there were significant differences in hospital admissions (p<0.001), length of hospital stay (p<0.01), and hospitalization costs (p<0.01).

**Table 2:** Comparison of medical utilizations among Groups I–IV. Group I (no smoking cessation or influenza vaccination), Groups II (influenza vaccination only), Group III (smoking cessation only), and Group IV (both smoking cessation and influenza vaccination)

*Group*	*Group I*	*Group II*	*Group III*	*Group IV*
**Emergency visits**, median (IQR)	2 (4)	0 (2)*	0 (2)*	0 (1)*
**Hospital admissions**, median (IQR)	1 (2)	0 (1)*	0 (1)*	0 (1)*^+^
**Length of hospital stay** (days), median (IQR)	7 (12)	0 (8)*	0 (9)*	0 (3)*^+^
**Hospitalization cost** (US$), median (IQR)	35422 (61474)	0 (28167)*	0 (41577)*	0 (12985)*^+^

IQR: interquartile range.

* vs Group I;

# vs Group II;

+ vs Group III. p<0.01.

**Figure 2:**
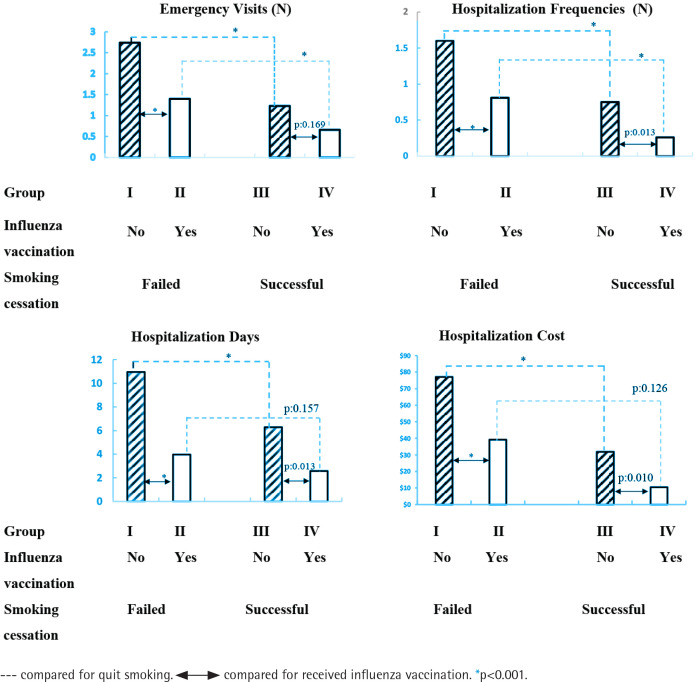
Medical utilization of emergency visits, hospital admission, hospital days, and costs in COPD patients among groups. Group I (no smoking cessation or influenza vaccination), Group II (influenza vaccination only), Group III (smoking cessation only), and Group IV (both smoking cessation and influenza vaccination)

As shown in Figure 2, successful smoking cessation groups showed a significant reduction in emergency visits (p<0.001) and hospitalization frequencies (p<0.001) compared to failed smoking cessation groups, both with and without influenza vaccination (Group III vs Group I, and Group IV vs Group II). Successful smoking cessation groups also showed a significant reduction in hospitalization days (p<0.001) and hospitalization costs (p<0.001) compared to failed smoking cessation groups without influenza vaccination (Group III vs Group I), but no significant reduction in hospitalization days (p=0.157) and hospitalization costs (p=0.126) compared to failed smoking cessation groups without influenza vaccination (Group IV vs Group II). Influenza vaccination groups showed significant reductions in hospitalization frequency (p<0.05), hospitalization days (p<0.05), and hospitalization costs (p<0.05) compared to non-influenza vaccination groups, both with and without successful smoking cessation (Group II vs Group I, and Group IV vs Group III). However, for influenza vaccination groups, there was no significant reduction in emergency visits (p=0.169) compared to the non-influenza vaccination groups, even with successful smoking cessation (Group IV vs Group III).

**Figure 3:**
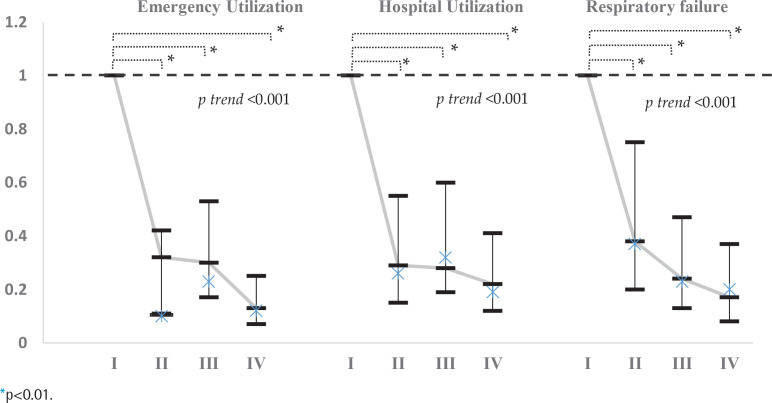
Odds ratios analysis of emergency utilization, hospital utilization, and respiratory failure in Groups II (influenza vaccination only), Group III (smoking cessation) and Group IV (both smoking cessation and influenza vaccination), compared to Group I (no smoking cessation or influenza vaccination), in addition, non-parametric tests for trend on emergency utilization, hospital utilization, and the occurrence of respiratory failure

Table 3 and Figure 3 present the trends in medical utilization, including emergency utilization, hospital utilization, and the occurrence of respiratory failure. Compared with Group I (neither smoking cessation nor influenza vaccination), Groups II, III, and IV had lower odds of utilizing emergency services, hospital utilization, and experiencing respiratory failure. Group IV (ex-smokers and influenza vaccination) had significantly lower odds ratios for emergency utilization (OR=0.13; 95% CI: 0.07–0.25), hospital utilization (OR=0.22; 95% CI: 0.12–0.41, p<0.001), and the occurrence of respiratory failure (OR=0.17; 95% CI: 0.08–0.37, p<0.001). Furthermore, the non-parametric tests for trends on emergency utilization, hospital utilization, and the occurrence of respiratory failure were all statistically significant (p<0.001).

**Table 3:** Odds ratios and 95% confidence intervals of the medical utilization and respiratory failure in Groups II–IV, compared to Group I. Group I (no smoking cessation or influenza vaccination), Groups II (influenza vaccination only), Group III (smoking cessation only) and Group IV (both smoking cessation and influenza vaccination)

*Variable*	*Emergency utilization*			*Hospital utilization*			*Respiratory failure*		
	*OR*	*95% CI*	*p*	*OR*	*95% CI*	*p*	*OR*	*95% CI*	*p*
Group I (Ref.)	1			1			1		
Group II	0.32	0.11–0.42	0.000	0.29	0.15–0.55	0.000	0.38	0.20–0.75	0.005
Group III	0.30	0.17–0.53	0.000	0.28	0.19–0.60	0.000	0.24	0.13–0.47	0.000
Group IV	0.13	0.07–0.25	0.000	0.22	0.12–0.41	0.000	0.17	0.08–0.37	0.000

## DISCUSSION

Our study demonstrates that the combination of smoking cessation and influenza vaccination has synergistic effects in reducing the medical burden of COPD. This includes a reduction in emergency department utilization, hospitalization utilization, hospitalization costs, and the occurrence of respiratory failure. The literature consistently shows that smoking is associated with a higher risk of hospitalization for influenza infection^13^.

Furthermore, smoking cessation and influenza vaccination can lead to overall cost reduction in medical expenses. In addition to the individual benefits of smoking cessation and influenza vaccination, quitting smoking may also improve immune function and enhance the effectiveness of the influenza vaccine.

Studies have shown that smoking can cause endothelial damage, thereby reducing the development of emphysema and vascular remodeling in COPD. Therefore, smokers have more comorbidities than non-smokers^7,18,19^. Smoking exacerbates inflammation, increases the susceptibility to infections, causes tissue damage, and may promote the development of allergies. Regarding infection risk, smoking impairs both the innate and adaptive immune systems, altering the function of various immune cells and signaling pathways^12,20^. For example, cigarette smoke reduces the ability of alveolar macrophages to effectively clear bacteria and cellular debris, leading to secondary inflammation^12,20,21^. Other innate immune cells, such as natural killer cells and dendritic cells are also affected by smoking^12,21^. Current and secondhand smokers have been found to have a higher risk of invasive pneumococcal diseases and community-acquired pneumonia compared to never smokers. Additionally, cigarette smoke can induce mitochondrial autophagy and programmed necrosis, which may contribute to inflammation and tissue damage. Maintaining a healthy immune system and restoring immune function are important strategies for improving COPD outcomes. However, COPD management and hospitalization are influenced by various factors^6,22^. For example, this study has shown that factors such as COVID-19, malignancy, and cardiovascular disease are associated with more extended hospital stays in COPD patients^23^. Previous research has also demonstrated that smoking cessation can prevent acute exacerbations of COPD, reduce incidence rates, improve survival rates^24^, alleviate respiratory symptoms, improve prognosis, and slow disease progression^25^.

Indeed, smoking is associated with lower rates of influenza vaccination among COPD patients. Current smokers are less likely to receive the flu vaccine compared to former smokers and never smokers^26^. It is essential for physicians to encourage current smokers to quit smoking and promote vaccination to prevent exacerbations and respiratory infections in COPD patients. A pilot study has shown that community pharmacists who provide patients with COPD with smoking cessation may increase their lives by 38.6 life years and 19.9 quality-adjusted life years^27^.

An impaired immune response to vaccination and infection in patients with COPD has been described^28,29^ . Patients with COPD may have impaired immune responses to vaccination and infection due to factors such as immune ageing, comorbidities, and the use of immunosuppressive agents, especially in the elderly population. However, studies on vaccine immunogenicity have demonstrated that influenza vaccination effectively induces immune responses in patients with COPD^26,28,30-32^. However, studies on vaccine immunogenicity have demonstrated that influenza vaccination is effective in inducing immune responses in patients with COPD^32^. Further research is needed to identify patients at risk of adverse reactions to vaccination and to develop improved vaccine strategies for specific populations.

Smoking also affects the adaptive immune system, leading to reduced antibody responses to various antigens and decreased serum levels of immunoglobulin classes. This can result in reduced T-cell function and increased production of autoantibodies^12,21,33^. Despite the lower influenza vaccination coverage among COPD patients compared to the Healthy People 2020 national goal^34^, studies have not found a statistically significant difference in the efficacy of influenza vaccination between smokers and non-smokers in the elderly population^11^. Additionally, no association has been observed between the response to influenza vaccination and current smoking status^35^. More research is required to further investigate the impact of smoking on the immunogenicity of influenza vaccination. Overall, while smoking can influence vaccination rates and immune responses in COPD patients, promoting smoking cessation and encouraging vaccination is important as part of comprehensive management strategies for COPD.

### Limitations

First, due to the study design, causal relationships cannot be established. Further studies with clinical outcomes are necessary to demonstrate the causality of the observed effects. Second, although many cases were initially considered, the study had to exclude a significant number of patients to ensure data integrity and account for factors such as age, routine lung function tracking, influenza vaccination time, and smoking status. The small proportion of included patients raises concerns about the representativeness of the findings to the entire COPD population. Third, the database used in the study was limited to one medical center, which restricts the generalizability of the results to other populations of patients with COPD. Variations in healthcare settings and patient demographics across different centers could impact the outcomes. Fourth, the impact of medical utilization in COPD patients is not solely determined by smoking cessation, influenza vaccination, or their combination. Other factors, such as socioeconomic status, ethnicity, quantification of current/previous smoking, and comorbidities, may influence the observed outcomes, but these were not fully accounted for in this study.

## CONCLUSIONS

Combined smoking cessation and influenza vaccination are more effective than either intervention alone or neither in reducing the medical burden associated with COPD, including hospital utilization, emergency utilization, and respiratory failure. However, further research is needed to investigate the effects of smoking, ageing, and comorbidities on the immunogenicity of influenza vaccines.

## Data Availability

The data supporting this research are available from the authors on reasonable request.

## PREPRINT

https://papers.ssrn.com/sol3/papers.cfm?abstract_id=4010879
